# Modeling Renal Disease “On the Fly”

**DOI:** 10.1155/2018/5697436

**Published:** 2018-05-31

**Authors:** Cassandra Millet-Boureima, Jessica Porras Marroquin, Chiara Gamberi

**Affiliations:** Biology Department, Concordia University, Montreal, QC, Canada

## Abstract

Detoxification is a fundamental function for all living organisms that need to excrete catabolites and toxins to maintain homeostasis. Kidneys are major organs of detoxification that maintain water and electrolyte balance to preserve physiological functions of vertebrates. In insects, the renal function is carried out by Malpighian tubules and nephrocytes. Due to differences in their circulation, the renal systems of mammalians and insects differ in their functional modalities, yet carry out similar biochemical and physiological functions and share extensive genetic and molecular similarities. Evolutionary conservation can be leveraged to model specific aspects of the complex mammalian kidney function in the genetic powerhouse* Drosophila melanogaster* to study how genes interact in diseased states. Here, we compare the human and* Drosophila* renal systems and present selected fly disease models.

## 1. Introduction

Defective kidney function can lead to potentially lethal end-stage renal disease (ESRD) and chronic kidney disease (CKD), for which therapeutic options are limited. ESRD and CKD may be remedied by dialysis and renal replacement therapy (renal transplant), which are both costly [1] and greatly affect the quality of life of both patients and their families. The complexity of the human kidney has posed a formidable challenge to experimental probing for many pathologies. In many cases disease progression is well described; however, the underlying mechanisms at the molecular and cellular levels are incompletely understood, which affects our capacity to design remedial therapeutics. Animal model research on kidney disease has traditionally used rodents for their mammalian-type kidney similar to the human one. However, rodent and human kidneys also share the same complexity, which challenges experimentation. Zebrafish, featuring a streamlined pronephros, has also been used to model renal disease, albeit less frequently, in part because of its adaptation to an aquatic environment. With an open circulatory system, the fly's renal system is aglomerular and urine formation is based on active transport rather than selective readsorption [2]. However,* Drosophila* is a clear evolutionary intermediate towards the glomerular kidney, with recognizable cell types responsible for fulfilling the kidney's main functions: detoxification, filtration, and endocytosis [3, 4]. The small body size and the fastest filtration rate known [5] allow flies to have separate compartments for renal function: the Malpighian Tubules (MTs), which are analogous to the renal tubules [6], and two clusters of nephrocytes within the body cavity, which are analogous to podocytes in the glomerular kidney. Because of extensive functional similarities, the fly has been successfully used to model aspects of mammalian renal function. We will compare the human and* Drosophila* renal systems and discuss the strategic use of fly modeling of human renal disease.

## 2. The Human Renal Filtration System 

The human kidneys, found in the mid to lower back of the trunk on each side of the spine, are bean-shaped organs roughly the size of a person's own fist. Composed of two main layers, the cortex and the medulla, they play a leading role in blood filtration, solute reabsorption, and metabolic waste excretion, which result in urine production. The kidney medulla contains so-called renal pyramids, conical regions collectively holding about one million functional units, which are called nephrons. The nephrons span both cortex and medulla, starting and ending in the former, with the latter containing variable lengths of the central portion of the tubule. Nephrons modify the filtered fluid and produce urine, which drains into collecting tubules (also called collecting ducts) that in turn fuse into larger ducts that empty into the minor calyx, the ureter and, eventually, the bladder.

**Figure 1:**
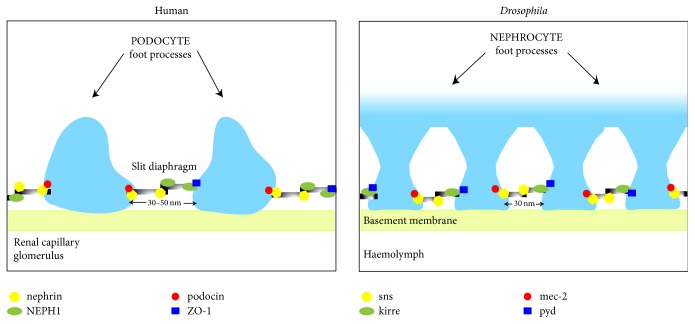
*Comparison of the human podocyte and the Drosophila nephrocyte slit diaphragms*. Selected evolutionary conserved proteins are indicated. The same symbols indicate orthologous proteins between human and* Drosophila*. In the fly, Duf was found to directly interact with Pyd and Sns with podocin ortholog Mec-2 [6].

**Figure 2:**
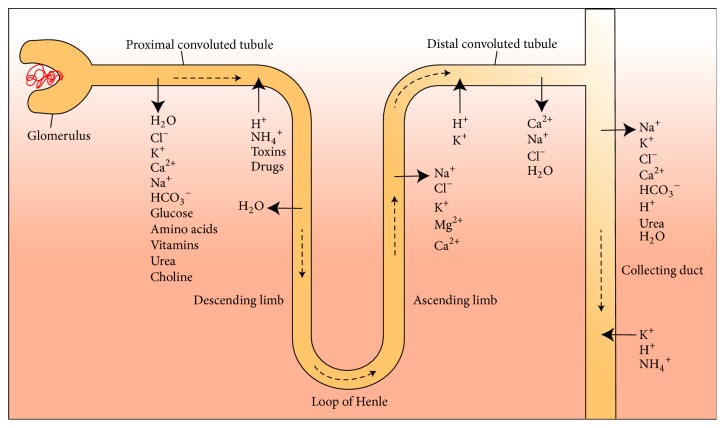
*Schematic features of a generalized human nephron*. The glomerulus, the different regions of the nephron, and corresponding ion and solute transport are indicated. The dashed arrows depict direction of the fluid flow.

Each nephron consists of a tubule closed at one end and enlarged into the cup-like Bowman's capsule, which surrounds a tuft of capillaries called glomerulus. Together, the Bowman's capsule and glomerulus are referred to as renal (or Malpighian) corpuscle. The renal corpuscle filters blood via specialized cells that respond to physiological cues. Glomerular capillaries are fenestrated, that is have pores which allow fluids and small molecules such as ions and sugars to leave the blood and, instead, retain cells and proteins exceeding pore size, complexes of carrier proteins and lipids, as well as calcium ions (Ca^2+^). Wrapped around the capillaries and with characteristic protrusions called foot processes which contact the capillary's basement membrane are podocytes, specialized epithelial cells integral to the filtration barrier [6–8]. Adjacent foot processes are separated by slit diaphragms about 14 nm wide with 30–50 nm wide pores carrying out filtration [6] (Figure 1). Major components of the slit diaphragm include members of the nephrin protein superfamily and NEPH1, which are coexpressed and form the diaphragm via homotypical and heterotypical interactions [6]. Together, the basement membrane, slit diaphragm, and podocyte processes form a barrier between plasma and filtrate which is essential to glomerular function. Its disruption can lead to kidney disease [9]. Differences in pressure between the glomerulus and Bowman's capsule determine the glomerular filtration rate (GFR, the amount of filtrate produced per minute), which is used to measure kidney function. Because ion and fluid balance depend on flow efficiency, glomerular filtration rate is subject to multiple regulatory mechanisms. The glomerular filtrate is first collected in the Bowman's capsule and directed through the nephron, flowing through the proximal convoluted tubule and the descending and ascending branches of the Loop of Henle, rising through the distal convoluted tubule while being modified, and, finally, arriving to the collecting duct as urine (Figure 2).

### 2.1. Human Nephron Development

Mammalian nephrons form during the late embryonic and early postnatal stages and display limited cell turnover, resulting in low regeneration rate in the adult [10]. Interactions between two mesoderm-derived tissues, the ureteric bud (UB), and the adjacent metanephric mesenchyme (MM) initiate nephrogenesis [10]. In part driven by developmental regulators Ret, Gfra1, Wnt11, Wnt6, and Pax2 in the UB and Bmp4, Gdnf, Pax2, and Wt1 in the MM, the UB invades the adjacent MM, generating the collecting duct [10–16]. The UB then branches to form a T shape within the MM. The two ends of the T structure then induce formation of the cap mesenchyme, which contains nephron stem cells and progenitors, as well as stromal cells that support kidney ontogenesis by producing signaling molecules, for example retinoic acid, promoting expression of Ret, ERK, MAPK, PI3K, PLC, and WNT [14, 17–20] in the UB. The iteration of this process produces both the branched structure of the ducts and nephron multiplicity. The MM cells then aggregate and, responding to Wnt signaling [21], undergo mesenchymal-to-epithelial transition to produce the renal vesicle or nephron progenitor [10–13]. Vesicle cells polarize, first establishing a proximal-to-distal axis [22] followed by an apical-basal one [23, 24]. After polarization, the renal vesicle reshapes in the form of a comma, then that of an S, and fuses with the UB, while cells differentiate morphologically [10, 25]. The S-shaped body gives rise to the glomerulus, Bowman's capsule, and both proximal and distal tubules. The intermediate region of the S-shaped body, instead, yields the loop of Henle [26]. Mesangial cells, integral to the glomerulus, derive from the stromal cells [15, 27]. Finally, the vasculature is formed by mesodermal cells that migrate into the developing kidney [28].

## 3. The* Drosophila* Renal System

In the fly, filtration is carried out by specialized cells called nephrocytes, which display remarkable similarities to the human podocytes [6]. Nephrocytes were originally discovered in the 1800s and found to uptake and store multiple compounds, including silver nitrate, albumin, and dyes [29–34]. Nephrocytes are found in two clusters: one, called pericardial, near the tubular heart and the other, called garland, harboring two nuclei, close to the esophagus [6, 32–36].* Drosophila* nephrocytes display characteristic in-folding of the plasma membrane which form channels flanked by foot processes [6]. Ultrastructural studies have revealed that nephrocytes form a three-layered filter morphologically similar to that formed by the vertebrate podocytes [6, 32–34, 36, 37]. Like human podocytes, pericardial nephrocytes filter and reabsorb solutes from the* Drosophila* circulating fluid, called haemolymph, via channels regulated by 30 nm wide slit diaphragms [38–40]. Slit diaphragms feature two filaments composed of proteins encoded by genes* sticks and stones* (*sns*) and* dumbfounded* (*duf*, also called* kirre*), a NEPH1 ortholog [6]. Other protein components of the nephrocyte slit diaphragm include the products of genes* mec-2*, a podocin ortholog, and* pyd*, a ZO-1 ortholog [6]. Alike human podocytes, a basement membrane enwraps each nephrocyte. Together, the nephrocyte diaphragm and the basement membrane form the filtration barrier in* Drosophila*, the integrity of which is maintained by protein-protein interactions between orthologs of the human slit diaphragm proteins (Figure 1). The filtrate is actively endocytosed from the sides of the channels, retained in cell vacuoles and either broken down (proteins) or stored (toxins, silver nitrate) [41]. The recent findings that nephrocytes may be apicobasally and basolaterally polarized reinforced their similarity with human podocytes [42, 43]. Moreover, nephrocytes and podocytes appear to respond similarly to pharmacological treatment. Administration of puromycin [44] and protamine sulfate [42] was found to disrupt the filtration barrier. Because of their extensive functional overlap with the necessary podocytes, the discovery that nephrocytes are, instead, dispensable in the adult fly, yet necessary for larval survival [45], was surprising and future studies are being targeted to understand this apparent paradox.

Nephrocytes have been used to model human nephrotic syndromes, in which podocyte processes are effaced as a consequence of mutations in the genes encoding for slit diaphragm proteins. Because these models have been recently reviewed, we refer interested readers to [46].

### 3.1. Malpighian Tubule Morphology

**Figure 3:**
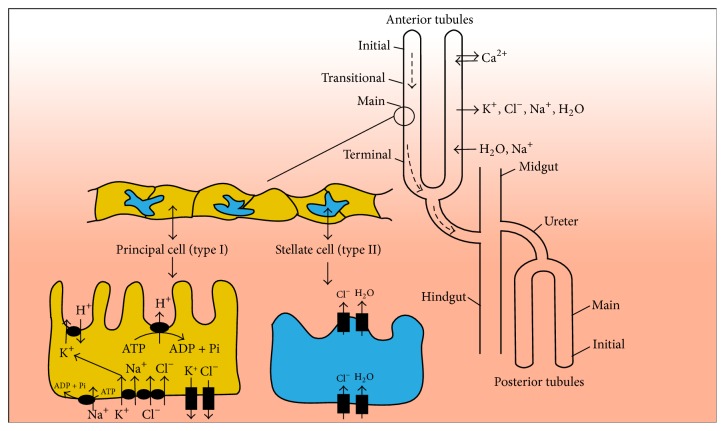
*Schematic features of the Drosophila Malpighian tubules*. The anterior and posterior tubules with relative functional segments and ion and water transport are indicated. Dashed arrows depict the direction of the fluid flow. Features and functions of principal and stellate cells are shown (below).

In* Drosophila* two pairs of MTs are made of a single-layered epithelium and depart from the interface between mid- and hind-gut (Figure 3). With one tubule residing more anteriorly and the other more posteriorly within the abdominal cavity, the MTs are folded in a stereotypical way, which is thought to ensure efficient metabolic waste removal and osmoregulation in the open circulatory system typical of insects. Unlike the mammalian closed circulatory system in which the circulating fluid is subject to glomerular ultrafiltration,* Drosophila* haemolymph is, instead, filtered. Also different from the mammalian nephrons that are embedded in organ tissues, the two MTs are free inside the fly's body cavity and can be cleanly microdissected. Anterior and posterior MTs can be distinguished both functionally and morphologically, because of their distinct transcriptomes and the anterior tubule being longer [47].

The anterior tubule pair can be divided into four sections: initial, transitional, main, and terminal (Figure 3). These regions contain type I cells, known as principal cells, and type II cells, known as stellate cells (Figure 3). Principal cells arise from a key interaction between the midgut and the hindgut, constitute about ~80% of all tubule cells, and are responsible for the transport of cations and organic solutes [48]. Stellate cells are scattered around the principal cells and are responsible for water and chloride ion (Cl^−^) flow [48, 49]. Reducing tubular expression of the vacuolar- (V-) ATPase by using fruit flies heterozygous for a lethal insertion in the gene encoding for the V-ATPase beta subunit revealed that cation transport may solely be performed by principal cells [50]. Stellate cells were only found in secretory regions and were absent from reabsorptive regions, suggesting that they may have secretory roles [50]. The cells in the MT initial segment are thinner and may excrete specifically Ca^2+^ ions at high rates [51]. Cells of the terminal segment appear to regulate ion and water balance via selective readsorption from and secretion into primary urine and by removing nitrogen-containing catabolites from the haemolymph via active transport of uric acid to the tubule lumen and passive diffusion of other molecules through the intercellular spaces. As the primary urine is transported along the tubule, it is sequentially transformed, a process that requires both apicobasal cell polarization of the tubular epithelium and planar cell polarity. Tubular cells were found to be functionally differentiated in a proximal-to-distal fashion and the processed urine is eventually secreted by the more distal cells. Because of their fundamental role in detoxification, the normal development of MTs is essential in the fruit fly.

Just like human nephrons,* Drosophila* MTs exhibit internal marked asymmetry which corresponds to distinct spatial domains of gene expression [50, 51]. Analyses of enhancer trap expression in the MTs revealed that the initial, transitional, and main segment of the anterior tubules and the sole main segment in the posterior tubules correspond to different cell types and distinct physiological functions [50]. The main segment was found to secrete potassium chloride (KCl) and water at high rates [52] and the lower third of the tubule carried out reabsorption [50]. The lower regions of the MTs appeared to modify the travelling fluid from the main segment by reabsorbing potassium ions (K^+^) [53] but not water [54], contrary to what was previously reported [50]. Moreover, the lower tubules were found to acidify the fluid and transport Ca^2+^ into the lumen (Figure 3) [53]. Remarkably, in just about 15 seconds, the cells located in the main segment of the* Drosophila* MTs were found to secrete fluid in amounts equal to their own volume, making the MTs the fastest known filtering system [52, 54]. While the observed functional complexity of the MTs was initially found surprising and in apparent contradiction with its reputation as a simplified epithelial developmental model [50], this same functional complexity turned out to be an asset for modeling human renal disease in combination with the available genetic and technological tools for probing ion transport [52].

### 3.2. Ion Transport and Fluid Secretion in the Malpighian Tubules

In the MTs, multiple ion transporters regulate ion balance in different sections. An apical V-ATPase generates a primary proton gradient that fuels the activity of sodium/proton (Na^+^/H^+^) and K^+^/H^+^ exchangers, also apically localized, which release Na^+^ and K^+^, respectively, in the lumen [54]. K^+^/Cl^−^ cotransporters localized at the basolateral membrane [55] decrease K^+^ concentration in the secreted fluid as it passes through the lower tubule [53]. Channels found in stellate cells transport Cl^−^ from the haemolymph to the lumen and are under control of the leucokinin peptide-hormone family [49, 54]. Leucokinins are synthesized in response to increased intracellular Ca^2+^ and promote both fluid secretion and epithelial permeability to Cl^−^ [56].

Cardioacceleratory neuropeptide CAP2b was found to stimulate fluid secretion specifically via cyclic GMP (cGMP) and to activate the nitric oxide (NO) signaling pathway [52, 57] that regulates salt and water balance in the fly MTs [58]. Early studies tested if increased concentration of intracellular Ca^2+^ could stimulate CAP2b and activate the NO/cGMP pathway in different cell types [59]. Producing the first Ca^2+^ reporter system in* Drosophila *MTs, Rosay and collaborators expressed aequorin, a Ca^2+^-sensitive luminescent protein, in principal cells in the tubule main segment via the GAL4/UAS binary expression system [59, 60]. As aequorin was produced in the tubules in vivo, luminescence indicated both the amount of aequorin and Ca^2+^ amounts. Stimulation of CAP2b-dependent physiological responses caused rapid Ca^2+^ release from internal stores [59]. Because in this system no CAP2b stimulation was observed in stellate cells, principal and stellate cells of the main segment are unlikely to be connected through gap junctions [59].

### 3.3. Malpighian Tubule Development

The MTs start forming as four primordia derived from the hindgut primordium and visceral mesoderm in the six-hour embryo [4, 61] in a process requiring the gap gene product Krüppel (Kr) and the transcription factor Cut [48, 62, 63]. The specification of future tubule cells is determined via Kr [48] and, similar to mammalian kidney development, the Wnt pathway [10, 48].

Each tubule primordium contains a unique tip cell specified by lateral inhibition via the Notch pathway [4, 48, 64]. The tip cell segregates and activates the Epidermal Growth Factor receptor homolog DER [65] which promotes cell proliferation, tubular growth, and development of the MTs excretory system [64, 65]. As the MTs grow closer to the caudal mesoderm they induce mesenchymal-to-epithelial transition in nearby cells that will insert themselves in the tubules and become stellate cells [4]. The ectoderm-derived tubular epithelium is formed of principal cells [4] and the ureter of ectodermal cells [4]. Cells divide a definite number of times to give rise to 146 principal and 33 stellate cells in each anterior tubule and 105 principal and 22 stellate cells in each posterior tubule in* Drosophila* [50, 66]. Most of tubule ontogenesis is completed during embryogenesis, and the MTs are not histolysed during metamorphosis. Using positively-marked mosaic lineage with GFP-labeled proliferating cells enabled the discovery of multipotent adult stem cells in the lower tubule and ureter [4]. Such cells require JAK-STAT signaling for self-renewal and are analogous to stem cells activated during repair of kidney ischemic injury [4].

### 3.4. Immune Function of the Malpighian Tubules

The MT epithelium is part of the fly's defenses against pathogens. The MTs display innate immunity with both humoral and cellular responses and no adaptive response, as is typical for insects [67, 68]. Remarkably, studies in* Drosophila* first revealed the immune function of Toll-receptor signaling [69]. In fact, the* Toll* gene, originally identified for its function in embryonic polarity [70], was later found to function in immunity [71] and to have a few homologs in the fly, including* “18-wheeler”* [72] and multiple vertebrate ones dubbed Toll-Like Receptors (TLRs). Unlike other fly organs involved in immunity, the MTs display constitutive production of antimicrobial peptides (AMPs) [73]. Upon sensing infection, the MTs activate distinct pathways when triggered by specific pathogens. The Toll pathway was found to respond to fungal and Gram-positive bacterial infections and the immune deficiency (IMD) pathway to respond to Gram-negative bacterial infections [74, 75]. MTs may also initiate a Toll-independent humoral response [76]. All these eventually trigger release of seven groups of AMPs, either directly from the MTs [73] or indirectly from the fat-body, the latter being the fly liver-equivalent [68, 69, 77]. The groups of AMPs, Drosomycin, Metchnikowin, Defensin, Attacin, Cecropin, Drosocin, and Diptericin, appear to inhibit growth of haemolymph-invading microorganisms [77]. Both the IMD and Toll pathways were found to sense superficial peptidoglycan on the bacterial cell wall via signaling by peptidoglycan-recognition proteins (PGRP) in the MTs principal cells and gut [69, 78, 79]. PGRP function has mainly been studied in the gut [80], yet the pathway appears to function similarly in the MTs. The Toll proteins display homology to the cytoplasmic domain of the vertebrate interleukin 1 receptor and participate into similar intracellular signaling cascades [81]. The IMD pathway is considered to be equivalent to the vertebrate TNF pathway [75]. Both Toll and IMD pathways result in activation of NF-*κ*B-like transcription factor Relish and induce transcriptional changes [82].

The steroid hormone ecdysone that regulates principal and stellate cell fluid secretion [68] also affects MT-dependent immunity. Ecdysone may promote haemocyte proliferation and fast pathogen encapsulation [83]. In S2 cells, ecdysone was also found to induce transcription of the* PGRP-LC* gene encoding the peptidoglycan receptor and, independently, of a subset of AMPs [84]. Ecdysone also triggers histolysis during metamorphosis [83]. However, the MTs are resistant to this process, possibly due to their fundamental role in immunity. Diap2, an antiapoptotic protein, was also found to contribute to the innate response in the IMD pathway, possibly via regulation of MT ion channels [85]. Diap2 levels increased in the MTs when there was an immune threat; conversely, decreased Diap2 made flies more prone to infections [85].

Finally, upon septic infection, the MT-dependent immune response may alternatively be activated via the NO pathway, which in turn initiates the IMD pathway and leads to increased NO Synthase (dNOS) and improved fly survival [67, 68].

With growing appreciation for the importance of the MT immune function, the ongoing mechanistic studies of gut-mediated immunity will provide resources and paradigms to better define the role of the MTs in the defense from pathogens.

## 4. Malpighian Tubules to Model Disease

MTs have been utilized to study the physiology of fluid transport because of their anatomical accessibility, streamlined anatomy, and one-cell-thick epithelium. In MTs, the proliferation of the founder cells (anlage), their spatial organization, patterning, and differentiation occur in sequence, rather than concurrently as in other epithelia, enabling studies of separate stages in time course experiments. MTs and mammalian nephrons share functionally distinct regions (Figures 2 and 3), analogous functions, and display remarkable transcriptome conservation [47, 86]. For example, similar to mammalian renal tubules, MTs carry out detoxification thanks to high levels of cytochrome P450 and glutathione transferase [87]. Likewise, mutations in evolutionarily conserved V-ATPase subunits were initially discovered in* Drosophila* because of their renal phenotypes [88, 89]. Three years later, equivalent mutations in the human* ATP6B1* V-ATPase were reported to cause similar defects in patients [90]. As the interest in modeling renal function in the fly continues to grow, we review some of the successful examples below.

### 4.1. Nephrolithiasis

*Drosophila* has been used to model the most common kind of human kidney stones, namely, calcium oxalate nephrolithiasis [91]. Nephrolithiasis refers to the formation and movement of kidney stones in the urinary tract [91]. There are multiple types of kidney stones that are distinguished for their different composition and origin. Largely dependent on diet and metabolism, kidney stones in the urinary tract are most commonly composed of calcium oxalate (CaOx) and, in lesser quantities, calcium maleate or phosphate. Also dependent on diet and metabolism, cysts composed of uric acid develop when urine is too acidic, for example in severe dehydration, gout, or following chemotherapy. Struvite cysts are caused by kidney infections and may result in urinary obstruction [91]. Finally, cystine stones form as crystals of leaked cystine in rare cystinuria patients. While rats had been the model of choice for CaOx stone formation [92], prohibitive costs of breeding and caring inspired Chen and colleagues [92] to model nephrolithiasis in the fly. Similar to rodents, flies appeared to respond to oral administration of lithogenic agents ethylene glycol, hydroxyl-L-proline, and sodium oxalate, by inducing formation of CaOx crystals in the MTs between two and three weeks after ingestion. Importantly, response severity was dose-dependent [92].

Recently, RNAi-mediated knockdown of the enzyme xanthine dehydrogenase* (Xdh)* in the fly was shown to induce ectopic calcification and accumulation of crystals and stones in the MTs [93]. Well-fed* Xdh-*knockdown flies only survived three days, as opposed to 60 days of the wild type control. Chemical analysis of the stones by micro X-ray fluorescence revealed significant amounts of Ca^2+^ and zinc (Zn). Because the latter had never been involved in kidney stone formation before, genetic confirmation was obtained by RNAi-mediated inhibition of Zn transporters in the fly, which was found to decrease stone formation [93]. Dietary and pharmacologic modulation of Zn levels in the fly and analyses of human kidney stones further confirmed Zn as a bona fide component [93]. In this case, the* Drosophila* model enabled the discovery of a new contributor to nephrolithiasis and indicated Zn-metabolic enzymes as potential therapeutic targets [93]. One issue to be clarified is that dietary Zn intake has been linked to increased risk of kidney stones in the adult (yet not in adolescent) individuals, while inhibiting Zn excretion was found to reduce cyst formation in the fly. One of the possible ways to interpret these apparently contradicting results posits that Zn may promote formation of different crystals depending on concentration [94] and indicates the need to probe additional physiological parameters in future studies to capture the complexity of kidney stone formation.

Flies have been used to study the processes leading to formation of uric acid stones because of their high levels of urate crystals normally accumulating in the tubule. Systematic analyses of the 33 genes encoding for subunits of the V-ATPase, some of which with multiple isoforms, revealed that mutants in the genes encoding core V-ATPase subunits displayed transparent MTs as a result of urine acidification, which decreased uric acid crystallization [89]. Notably, similar acidification defects were also found in patients with mutations in two V-ATPase subunits, which suggests a certain degree of functional conservation [90, 95].

### 4.2. Polycystic Kidney Disease

Polycystic kidney disease (PKD) is a genetic disease affecting at least 12.5 million people world-wide, regardless of ethnicity [96]. Two forms of PKD exist, one autosomal dominant (AD) and one, rarer and more severe, which is autosomal recessive (AR) [96] and will not be discussed here. ADPKD causes the development and progressive enlargement of fluid-filled cysts in the nephron, that consequently increase kidney size and cause interstitial fibrosis and chronic kidney disease by age 55 [96]. In half of the patients the severe damage results in kidney failure, making dialysis or renal transplant the only treatments [96]. The lack of a cure and dialysis costs that can surpass 150,000$ per patient per year [1] make PKD a global priority.

*Genetic Underpinning of PKD. *More than 85% of ADPKD patients carry mutations in the* PKD1* gene, which encodes polycystin-1, a G-protein coupled receptor (GPCR) [97]. Complete mutational inactivation of both alleles is rare and lethal pre- or peri-natally [96]; however, incompletely penetrant* PKD1* alleles have been found in homozygosis [98]. Mutations in another gene,* PKD2*, are found in about 10% of ADPKD cases [97].* PKD2* encodes polycystin-2, a transmembrane calcium channel of the TRPP family which was found to physically interact with polycystin-1 [99, 100]. The remaining ~5% of ADPKD patients carry unknown mutations other than* PKD1* or* PKD2* [101]. Because of their clear implication in PKD etiology,* PKD1* and* PKD2 *genes and corresponding polycystin-1 and polycystin-2 gene products are being studied in much detail. Polycystin-1 and polycystin-2 complexes were found to mediate cell-matrix and cell-cell interactions, planar cell polarity, signal transduction, and cilia-mediated mechanosensation [96]. We have recently reported that cystic tissue from ADPKD patients carrying a* PKD1* mutation exhibited significant reduction of the* Bicaudal C (BICC1)* gene expression [102]. Similarly,* Pkd1*^−/−^ mice displayed reduced Bicc1 protein specifically in the kidneys [102], placing* BICC1* genetically downstream of* PKD1*. Mutations in the* BicC* gene of many vertebrates, including humans, cause the development of renal cysts [103–110].* BicC* was originally discovered in the fly during a screen for embryonic polarity determinants in the germline [111].

Cyst formation is complex and unfolds over time. ADPKD patients carrying* PKD1* or* PKD2* mutations already display small renal cysts at birth [96] yet remain asymptomatic until middle age because the renal capacity is in vast excess (in fact, donation of one kidney is compatible with life). After then, kidney function declines rapidly. Because polycystin-1 and polycystin-2 are part of multiple protein complexes with wide cellular distribution, dysregulation of either in the renal tubule affects many pathways, including apicobasal and planar cell polarity, cell proliferation, cell metabolism, fluid secretion, and the extracellular matrix [112–115]. In PKD cysts form in the renal tubular epithelium where some cells reactivate normally quiescent proliferation pathways and begin to divide. In parallel, epithelial polarization is progressively lost, impacting secretion. Fluid accumulation in the cysts, in turn, stimulates further cell division, possibly in response to increased tensional stretch in the tissue [116, 117]. It is currently unclear what triggers cyst formation. As cysts expand, tubular cells display activation of various signal transduction pathways mediated by Ca^2+^ and cAMP, e.g., Raf-MEK-ERK [118, 119], the mammalian Target of Rapamycin (mTOR), PI3-kinase-Akt, JAK-STAT, NF-kB, Wnt, Hippo, and G-proteins [115, 118–130]. In spite of the enumeration of these pathways, the mechanisms of cyst initiation and progressive cystic degeneration remain largely unknown, likely because of the anatomical complexity of the vertebrate kidney and slow disease onset, which hinder experimental probing. The* BicC* fly provided the first account of renal cysts in* Drosophila* [102]. Modeling PKD in the fly may enable biochemical characterization of the cystic tubule and define the genetics of cyst formation and progression due to low genetic redundancy, and may advance our understanding of the core cystic processes.* BicC* encodes a conserved cytoplasmic RNA-binding protein with orthologs in many species [104, 105, 109, 131–133]. BicC can bind to multiple mRNA targets and appeared to reduce their expression posttranscriptionally [134]. The resulting target upregulation in the oocytes from heterozygote* BicC* female flies was found to disrupt anterior-posterior embryonic polarity [7, 115–135], while* BicC *homozygotes displayed oogenesis arrest at stage 10 [136]. Similar to ADPKD patients,* BicC* mutant flies featured fluid-filled cysts in the MTs already at hatching; over time the cysts enlarged and became more numerous [102]. Compared to wild type,* BicC* flies were short-lived, possibly a consequence of their defective renal function [102].* BicC* MTs also displayed extra branches, indicating underlying developmental and polarity defects. Oocytes from* BicC* mutant flies exhibited abnormal actin structures which prevented secretion of the dorsal fate determinant Gurken [137–141]. Similarly, the BicC protein was required for epithelial polarization via cadherin-mediated cell adhesion in the IMCD murine kidney cell line [142]. Initial molecular analyses of the* BicC* MTs identified the activation of the* myc* and TOR pathways, two hallmarks of vertebrate PKD [102]. Like ADPKD patients, postrenal transplantation (in which diseased kidneys are left in place) and administration of the immune suppressant and TOR inhibitor rapamycin could transiently rescue the* BicC* flies and reduce cysts, relative to untreated controls [102, 126]. Murine PKD models also exhibited mTOR cascade stimulation [143–148] and responded to rapamycin by delaying cystic onset [126, 145, 149]. In sum, the* BicC* cystic flies appeared to recapitulate many of the diseased features of PKD, displayed pharmacological response to rapamycin [102], and may be a valid model to advance our understanding of the molecular bases of renal cyst formation and the formation of extra tubular branches. One interesting aspect is that ciliary (dys)function appears prominent in vertebrate PKD [150]. The absence of ciliated epithelia in* Drosophila* raises the intriguing question of how cysts form and develop in* BicC* MTs versus human nephrons. Considering that other ciliary pathways, e.g.,* hedgehog*, were originally discovered in the fly, the striking biochemical similarities between PKD-type cysts and the* BicC*-dependent cysts in the fly may not be as surprising and may suggest new hypotheses on the evolution of ciliary function.

With proper consideration of the differences between flies and humans and of the hierarchical relationship between the* BicC* and* PKD1* genes, the* BicC* cystic fly may offer opportunity to chart conserved pathways that are altered in* BicC* mutation, are relevant for cyst formation and/or progression, may allow to form new hypotheses on* BicC* function and disease mechanism, and contribute to our understanding of the larger functional context of human PKD. Considering that BicC was also found in a protein complex linked to human nephronophthisis, another cystic kidney disease [110, 133], future studies will reveal if* BicC* function may affect multiple pathways of renal cystogenesis.

## 5. Conclusion

The remarkable conservation of renal functions between fruit flies and humans is suggestive of the presence of strong evolutionary constraints imposed on the detoxification process of all organisms. Emerging evidence of the interplay between renal and immune functions suggests additional requirements for the renal system. Multiple diseases causing progressive degeneration and loss of function of the kidney result in organ damage that may only be remedied by renal replacement therapy or dialysis, which are costly socially for the health care system and personally to the patients and their families, due to their negative impact on quality of life. Studies aiming at understanding the mechanisms of renal disease have been hindered by the anatomical complexity of the mammalian kidney.* Drosophila* possesses an evolutionary intermediate between glomerular and nonglomerular renal system, consisting of anatomically separated renal tubules and nephrocytes that, together, fulfill the renal functions. Similar developmental origin of the fly MTs and nephrocytes with their human counterparts, the nephron and the glomerular podocytes, respectively, is accompanied by conserved cellular pathways. Making the fruit fly a useful model to study the mechanisms of disease, the structurally streamlined, anatomically isolated, renal structures can be easily microdissected and studied biochemically; moreover, they can be probed genetically utilizing the vast array of* Drosophila* genetic tools. In multiple cases in which human renal disease has been modeled in* Drosophila*, including nephrolithiasis and PKD, the conservation seemed to extend to pharmacological responses, echoing similar examples in other fly disease models. Considering that many drug binding sites were found to be conserved in the fly [151], development of proper pharmacological screen protocols in the fly may in future provide a rapid and effective alternative strategy for drug discovery.
